# A Case Report of an Intradural Extramedullary Tumor, Which Was Treated With Steroid and Clinically Suspected as Neural Sarcoidosis

**DOI:** 10.1155/cro/9401516

**Published:** 2026-06-07

**Authors:** Shogo Suenaga, Kenji Kato, Kiyoshi Yagi, Hideki Murakami, Nobuyuki Suzuki

**Affiliations:** ^1^ Department of Orthopaedic Surgery, Nagoya City University Graduate School of Medical Sciences, Nagoya, Aichi, Japan, nagoya-cu.ac.jp

**Keywords:** intradural extramedullary tumor, neural sarcoidosis, steroid

## Abstract

Sarcoidosis is a systemic disease that is represented by lymph node swelling of the lung with pathologically diagnosed nonfibromatous necrosis. We report a rare case that was clinically diagnosed as an intradural extramedullary tumor of sarcoidosis, which was treated with oral steroid medication. A 45‐year‐old woman visited a local clinic due to leg pain and muscle weakness. MRI showed an intradural extramedullary tumor at the level from Th 4 to 5. The radiologist′s comment on MRI suggested that the tumor was a meningioma. She was referred to our hospital for emergency surgery due to muscle weakness. On administration, the muscle strength was Grade 4 on manual muscle testing and no bladder or rectal disorder was observed. The appearance of the tumor was not typical for the diagnosis of meningioma and the prior diagnosis could be neural sarcoidosis or a hematologic tumor as differential diagnoses. Lab data and bone marrow aspiration did not prove a hematologic tumor. CT revealed bilateral hilar lymphadenopathies, whereas pathological examination by bronchoscopy did not show evidence of sarcoidosis, such as caseating granuloma. Since resection of intradural tumors carries a high risk of complications, the mass was clinically suspected as sarcoidosis and treatment of the tumor with steroid started. After treatment, the pain and muscle weakness diminished soon, and 2 months later, the tumor clearly disappeared. In the follow‐up period, she also suffered heart sarcoidosis and continued steroid therapy for heart problems. If intradural extramedullary sarcoidosis was diagnosed before surgery, patients do not need surgery. It is important for surgeons to know this disease.

**Key Points**

• Intradural extramedullary tumors can be neural sarcoidosis.
• Neural sarcoidosis can be treated with steroids.
• Surgical treatment can be avoided if the tumor is diagnosed prior to surgery.

## 1. Introduction

Sarcoidosis is one of the systemic diseases that is treated with steroid medication. Although sarcoidosis is widely known, the diagnosis of sarcoidosis is not standardized. The official American Thoracic Society clinical practice guideline stated that there are three major criteria for diagnosis: a compatible clinical presentation, the finding of nonnecrotizing granulomatous inflammation in one or more tissue samples (not always required), and the exclusion of alternative causes of granulomatous disease. However, they also stated that there are no established objective measures to determine if each of these diagnostic criteria has been satisfied, and, therefore, the diagnosis of sarcoidosis is never fully secure [1].

Among spinal tumors, neural sarcoidosis is also included as one of the intradural extramedullary spinal tumors [2–4]. The reviews indicated that neural sarcoidosis is most common in the cervical spine. Most reports suggested that surgical treatment is successful. However, since sarcoidosis is a systemic disease, patients may need steroid treatment to control other lesions. Therefore, if a tumor is diagnosed as neural sarcoidosis, steroid treatment should be considered to avoid unnecessary surgery. Although neural sarcoidosis could occur in intramedullary lesions [5], not in extramedullary lesions, which cannot be diagnosed by biopsy because of safety and are treated with steroids. We report that the patient who was successfully treated with steroids fully recovered from her pain and muscle weakness due to an intradural extramedullary tumor, which was clinically suspected as neural sarcoidosis.

## 2. Case Presentation

A 45‐year‐old woman felt a decrease in sensation and numbness in her right lower leg. After 5 months of initial symptoms, she visited a local orthopedic clinic and received medication such as vitamin B12, as suggested, probably due to cervical spine or lumbar spine problems. After another 2 months, she felt weakness in the lower leg muscles and visited the neurology clinic. The brain magnetic resonance image (MRI) did not show any evidence of brain problems such as a tumor or cerebral infarction. Finally, she underwent thoracic and lumbar spine MRI and was diagnosed with an intradural extramedullary tumor in the thoracic spine. Then, she was transferred to our hospital for the possibility of urgent surgery due to muscle weakness.

On admission, the manual muscle test (MMT) was assessed as iliopsoas 4/5, quadriceps 5/5, tibia anterior 3/5, calf 5/5 (R/L), and tendon reflex was as PTR +++/++ ATR ++/++ (R/L), and ankle clonus was bilaterally positive. All upper arm tests were normal. These findings, which were muscle weakness and bilateral spasticity, matched a thoracic lesion. She had not yet suffered bladder or rectal disorder.

MRI revealed that there was a horseshoe‐shaped mass in the spinal canal at the thoracic 4–5 level. The mass looked intradural extramedullary tumor. The tumor showed intermediate signal intensity by T1‐weighted images and hypointensity by T2‐weighted images and the tumor was clearly enhanced by gadolinium injection (Figure 1). Although the report by a radiologist said that the tumor looked meningioma, we thought the tumor looked like a hematologic tumor or inflammatory mass such as sarcoidosis rather than meningioma because the expansion of the shape looked that tumor would be soft. Computed tomography (CT) was assessed and found a bilateral hilar mass. The report by a radiologist suggested the hilar mass would be lymphadenopathies or another tumor, and did not mention sarcoidosis as the first diagnosis. The mass was assessed by hematologists to see whether the tumor would be a type of lymphoma. There was no evidence of hematologic tumor by lab data and bone marrow aspiration. The blood test results were followed, the values in parentheses are reference values. IgG4; 47.5 mg/dL (11.0 ~ 121.0), beta‐2 microglobulin (*β*2‐MG); 2.5 *μ*g/mL (0.8 ~ 1.8), angiotensin‐converting enzyme (ACE); 11.4 U/L (8.3 ~ 21.4), soluble interleukin‐2 receptor (sIL‐2R) sIL‐2R; 425 U/mL (122~496). A bilateral hilar mass was also assessed by a biopsy for pathology examination by bronchoscopy, but it resulted in no evidence of sarcoidosis, such as a caseating granuloma. Other systemic assessments were performed to see if the mass fit the criteria for sarcoidosis, but there was no evidence of heart, eyes, or skin problems, which are other major organs of sarcoidosis. With these, sarcoidosis could not be diagnosed by the criteria [1]. During these assessments, she continued admission in our hospital to be carefully observed for her symptoms, and her symptoms did not worsen. After these assessments, the only way to diagnose this tumor was spinal surgery. As there were not a few risks due to spinal surgery, especially an intradural tumor, it could be one option to treat this tumor by steroids before the surgical procedure. After careful discussion, on Day 26 after admission to our hospital, even without a biopsy, the mass clinically suspected as sarcoidosis and started treatment with steroid medication. Since admission, her numbness and weakness of legs had not changed at all; however, her numbness and muscle weakness clearly improved just 2 days after steroid treatment. The first amount of steroid was 45 mg/day of prednisolone, and the amount tapered a half dose every 2 weeks. Her muscle weakness fully recovered 10 days after steroid treatment. MRI was performed 2 months later of the steroid, showing that the mass clearly became smaller (Figure 2)a. The amount of steroid continued to reduce and finished the medication after 5 months.

**Figure 1:**
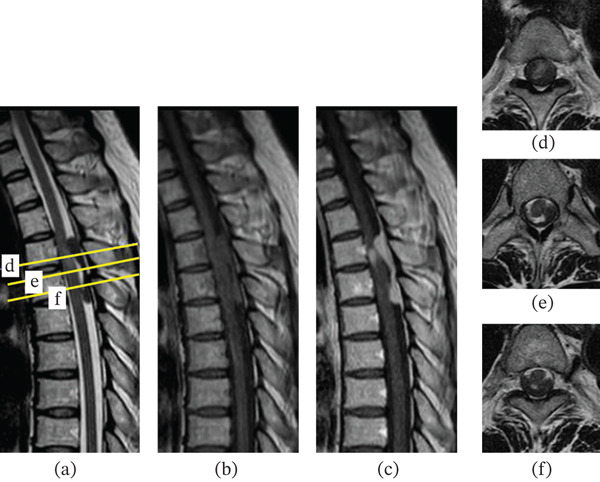
MRI at the first visit. (a) A sagittal image of a T2‐weighted image. A tumor was seen at the T4‐5 level. Yellow lines show axial levels corresponding to d, e, and f. (b) A sagittal image of the T1‐weighted image. (c) A sagittal image of a gadolinium‐enhanced T1‐weighted image. (d–f) Axial images of the T2‐weighted image.

**Figure 2:**
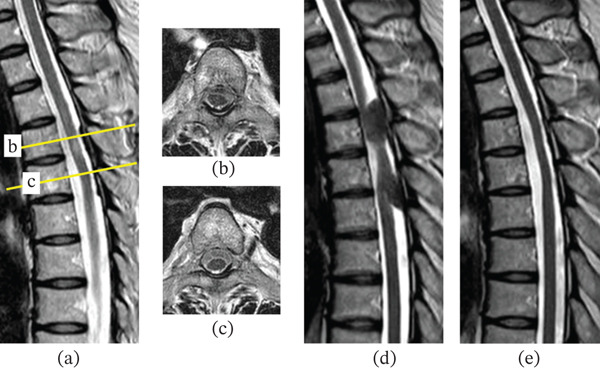
(a) A sagittal image of a T2‐weighted image after 2 months of steroid treatment. The tumor disappeared. Yellow lines show axial levels corresponding to b and c. (b, c) axial images of the T2‐weighted image. (d) MRI after 1 year of follow‐up. (e) MRI after 4 months of readministration of the steroid.

One year after the first visit, the follow‐up MRI showed the recurrence of the mass at the same place (Figure 2d), and prednisolone (10 mg/day) was started again while she did not have any symptoms. After 4 months of readministration of steroids, the tumor again clearly disappeared (Figure 2e). Because she had side effects such as weight gain and moon face appearance, the steroids were reduced and finished after tapering again.

Two years after the first visit, a follow‐up MRI again showed the recurrence of an intradural mass, while she did not feel any myelopathy symptoms. At the same time, she was clinically diagnosed with cardiac sarcoidosis because of pericardial effusion. After discussion with cardiologists, steroid treatment was administered at 5 mg/day of prednisolone. However, pericardial effusion increased, and a dose of prednisolone was increased by cardiologists to 30 mg/day. With steroid therapy, the mass again disappeared within 4 months. Thereafter, steroid therapy is controlled by cardiologists, and there has been no recurrence of intradural mass, and she remained free of spinal cord‐related symptoms. After more than 6 years of follow‐up, she reported no pain or numbness, and MMT of her lower extremities is 5/5.

## 3. Discussion

Since spine surgeons do not often treat patients with neural sarcoidosis, it is not easy to diagnose patients as sarcoidosis. We understand that diagnosing our case as sarcoidosis was also debatable. Even though she suffered bilateral hilar mass, which is a typical finding of sarcoidosis, the result of the biopsy did not show nonnecrotizing granulomatous inflammation. Furthermore, we assessed the eyes, heart, and skin to see other compatible findings of the criteria. However, initially, there were no suggestions of sarcoidosis. After careful discussion, we decided to use a steroid to treat the tumor.

There are several reports about intradural extramedullary tumors that were diagnosed as sarcoidosis. All of those were diagnosed after surgery, and they reported a satisfied course of patients even with paralysis before surgery [6, 7]. Connor et al. [8] reported the MRI findings of a spinal intradural extramedullary sarcoid mass. However, at that time, they found only five reports and concluded no typical findings of sarcoidosis compared with schwannomas and meningiomas, which are the commonest intradural extramedullary tumors, such as T1‐intermediate or hypointensity and T2 hyperintensity signals. After their report, some case reports have been published (Table 1) [3, 7, 9–12]. There are not yet typical features of intradural extramedullary sarcoidosis. Ishiwata et al. [12] reported a case report in 2019. The features of MRI in their report were similar to our case. The features of their case look like a horseshoe shape or rosary‐like shape, and a little long vertically. On the other hand, most schwannomas and meningiomas are usually round‐shaped. They commented that even though they discussed intensity or a dural tail sign of mass and other reported features of sarcoidosis, they could not diagnose it before surgery. They did not use steroids after surgery because there was no evidence of systemic sarcoidosis and no recurrence of spinal mass. When steroid therapy is effective, and symptoms reduce even for lung sarcoidosis, it is acceptable to finish steroid therapy [13]. Therefore, it is reasonable that they did not use steroids after surgeries.

**Table 1:** Clinical features in recent cases of intradural extramedullary sarcoidosis.

	Age/female or male	Symptoms	Location	MRI findings	Initial diagnosis	Reference
1	33/F	Neck and right arm pain, muscle weakness	C5	T1 intermediate, T2 high and well enhanced	Neurofibroma	Bose 2002 [9]
2	42/F	Numbness in hands and feet	C5‐6	T1 intermediate, T2 low and well enhanced	Meningioma	Hamasaki 2003 [3]
3	30/F	Neck and muscle weakness of lower limbs	C7‐1	T1 intermediate, T2 mixed and well enhanced	Meningioma	Roy 2010 [10]
4	38/M	Not described	C5‐6	T1 intermediate, T2 high and well enhanced	Not described	Radwan 2017 [11]
5	32/M	Numbness and paresthesia of the right hand, trunk, and both lower limbs	C3‐4	T1 intermediate, T2 low and well enhanced	Meningioma	Ishiwata 2019 [12]
6	29/F	Numbness and paresthesia of left lower extremity	T8	T2 intermediate and well enhanced	Not described	Shields 2021 [7]

Roy et al. reported a case report and literature review [10]. We would like to pick up an important message from their report, which is that their case developed recurrence after 6 months and the patients took steroid therapy. They concluded that the natural history of spinal sarcoidosis is remission and relapse, and corticosteroids are the cornerstone of continuing medical treatment. This could suggest that if patients were diagnosed with sarcoidosis and started to take steroids before surgery, they would not need to undergo surgery.

In our case, initially, a surgery to resect a mass by posterior decompression was one option. To decide a diagnosis, we discussed that it was necessary to perform a biopsy of the mass by surgery, too. As we know, there are severe surgical complications such as infection, spinal fluid leakage, or paralysis. Therefore, we clinically suspected her as neural sarcoidosis and started steroid therapy without biopsy of the intradural extramedullary mass. As we expected, the mass completely disappeared after steroid therapy. In addition, later, she suffered heart sarcoidosis and needs to continue steroid therapy (5 mg/day of prednisolone), either way. At this moment, the bilateral hilar mass does not change, and she does not have any lung symptoms.

Other differential diagnoses for intradural extramedullary tumors include Rosai–Dorfman disease (RDD), Erdheim–Chester disease (ECD), IgG4‐related disease (IgG4‐RD), and Langerhans cell histiocytosis (LCH). RDD is a subtype of non‐LCH that primarily affects the skin, subcutaneous tissues, and lymph nodes [14]. Although rare, spinal involvement in RDD has been reported [15], typically presenting as a relatively diffuse intradural mass. ECD, once considered an inflammatory disorder, is now recognized as a hematopoietic neoplasm [16]. Clinical features of ECD and RDD can sometimes overlap. ECD manifests in diverse organ systems, including the aorta, heart, brain, bone, lungs, and skin; its intradural presentations often mimic meningioma [17]. IgG4‐RD is an uncommon fibro‐inflammatory condition characterized by lymphoplasmacytic infiltration, obliterative phlebitis, and fibrosis across various organs [18]. Spinal lesions in IgG4‐RD range from hypertrophic pachymeningitis to focal intradural masses, making radiologic diagnosis challenging [19, 20]. LCH is a myeloid neoplastic disorder most frequently involving the bone [21, 22]. It is more common than RDD, ECD, or IgG4‐RD, with the systemic form predominating in children. Although LCH typically affects the spinal vertebrae, it can also occur within the spinal canal, so that LCH can also be included in the differential diagnosis of intradural masses [23]. The clinical and radiological characteristics of these differential diagnoses are summarized in Table 2.

**Table 2:** Differential diagnosis of intradural extramedullary tumors.

Disease	Primary involved sites	Radiological or clinical key features
Sarcoidosis	Lungs, lymph nodes, and eyes	Often multifocal; responds to steroids; “clinically suspected” in this case.
Rosai–Dorfman disease	Skin and lymph nodes	Relatively diffuse intradural masses; non‐Langerhans cell histiocytosis.
Erdheim–Chester disease	Heart, bone, and brain	Often mimics meningioma; hematopoietic neoplasm.
IgG4‐related disease	Multiple organs	Hypertrophic pachymeningitis or focal masses; fibro‐inflammatory.
Langerhans cell hisitocytosis	Bone (primarily)	More common in children; typically vertebral but can be intradural.

To date, there are no reports about steroid‐treated intradural extramedullary tumors of sarcoidosis with good results without surgery. Currently, there are no established diagnostic criteria for intradural sarcoidosis. We hope that accumulation of similar case reports will facilitate the development of such criteria in the future. We here report a case of an intradural extramedullary mass that was clinically suspected as sarcoidosis and treated successfully with steroids. If a mass looks unusual for schwannomas or meningiomas, it is important to consider neural sarcoidosis to avoid unnecessary surgeries.

## 4. Limitation

The major limitation of this case was the absence of a biopsy and there was no pathological evidence of sarcoidosis. A biopsy of the intradural mass at particularly thoracic spinal cord level was deferred due to the significant risk of severe complications. Consequently, the diagnosis was established based on clinical presentation and characteristic radiological findings, although it remains “clinically suspected.”

## 5. Conclusion

We report a case of clinically suspected intradural extramedullary tumor of neural sarcoidosis, which was successfully treated with steroid medication without surgery. With this disease in mind, unnecessary spinal surgeries can be avoided.

## Funding

No funding was received for this manuscript.

## Ethics Statement

Informed consent to allow publication was obtained from the patient described in this report. This case report was prepared in accordance with the CARE guidelines.

## Conflicts of Interest

The authors declare no conflicts of interest.

## Data Availability

Data sharing is not applicable to this article as no datasets were generated or analyzed during the current study.
